# P-1242. Missing big in our little patients: Vancomycin trough concentrations do not predict IDSA guideline targeted AUCs

**DOI:** 10.1093/ofid/ofae631.1424

**Published:** 2025-01-29

**Authors:** Marc H Scheetz, Justin Shiau, Paul Sabourenkov, Sharmeen Roy

**Affiliations:** Midwestern University, Downers Grove, Illinois; Midwestern University Pharmacometrics Center of Excellence, Downers Grove, Illinois; DoseMe Pty Ltd, Brisbane, Queensland, Australia; DoseMe, Miami, Florida

## Abstract

**Background:**

Therapeutic drug monitoring is the guideline-recommended, standard of care for vancomycin because of the known efficacy and safety exposure window [i.e. area under the concentration curve (AUC) of 400-600 mg*24hours/L]. Yet, surveys show that few practitioners calculate AUCs because of the perceived adequacy of trough concentrations; however, the percentage of real-world pediatric patients with goal vancomycin trough concentrations that achieve target vancomycin AUC remains unknown. Thus, we queried a large cohort of internationally represented, real-world, pediatric patients with precise estimates of vancomycin AUC exposures and measured trough concentrations.

**Figure:**
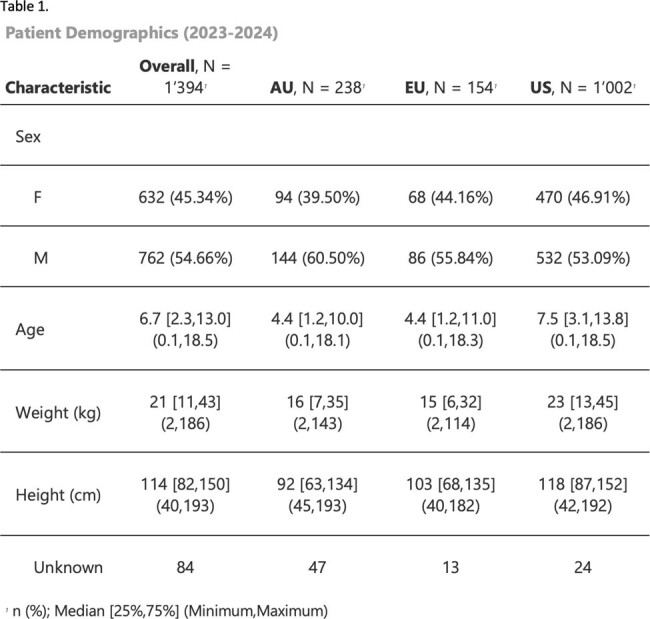

**Methods:**

Pediatric patients treated with vancomycin between 2023-2024 and with therapeutic drug monitoring (TDM) performed had data anonymized via an electronic clearinghouse. Unique patients and dosing events were quantified; trough concentrations within 2 hours of the next dose were identified, and patient individualized AUC was calculated using a Bayesian method with 2 validated models. For each dosing event, pairs of trough and AUC24 were compared and categorically classified as “low”, “target”, and “high” according to trough and AUC (i.e. 15-20 mg/L and 400-600 mg*24hours/L, respectively). Prediction intervals (90%PI) were calculated.

**Figure 1.**
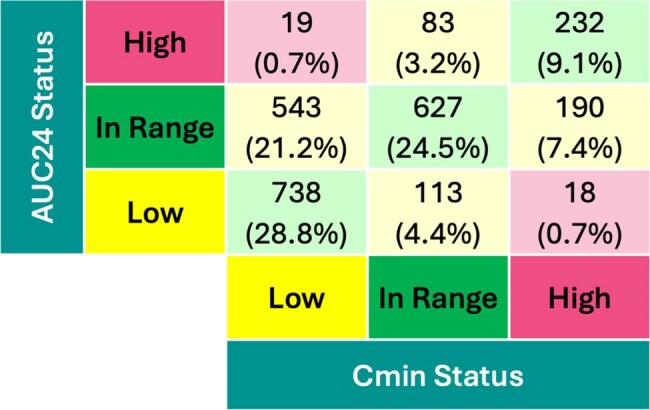
Categorical agreement of troughs and AUC between "low, in range, and high"

**Results:**

A total of 1,394 pediatric patients from the United States (72%), Australia (17%), and the European Union (11%) with 2,563 dosing events with paired trough and AUC were included (Table 1). Categorical disagreement between trough and AUC was 37%, most disagreement (2,563 dosing events, 21.2%) occurred at low trough but AUC at target. Only 24.5% of paired trough and AUC were simultaneously at target. AUC was highly variable for all trough categories (i.e. low, target, and high, Figs. 1,2). Troughs of 15 and 20mg/L resulted in a AUCs with 90%PIs of 334-725 and 419-811 mg*24hours/L (Fig. 3), respectively.

**Figure 2.**
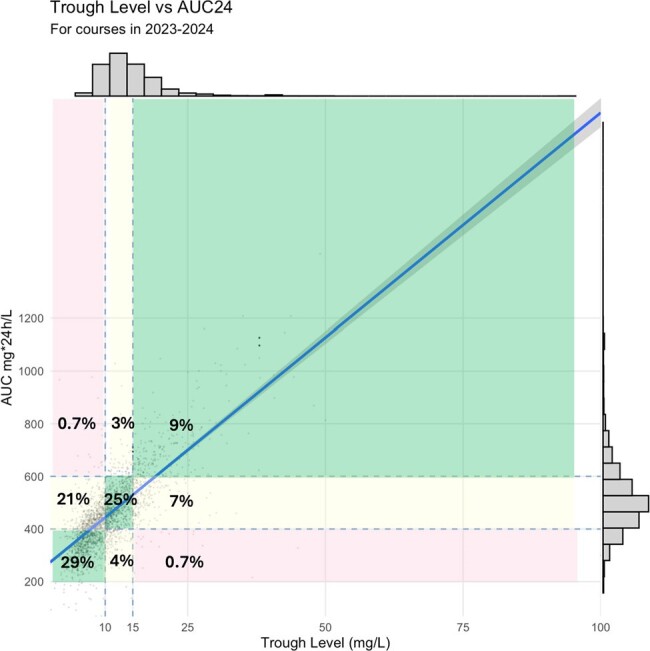
Scatterplot of trough by AUC with categorization overlaid

**Conclusion:**

Trough concentrations resulted in frequent misclassification of AUC category, with troughs< 15 mg/L frequently resulting in AUCs >400 mg*24h/L. These findings are important as low troughs result in additional unnecessary sampling, compel clinicians to increase doses, and lead to unintended high AUCs. Importantly, high AUCs cause increased kidney injury.

**Figure:**
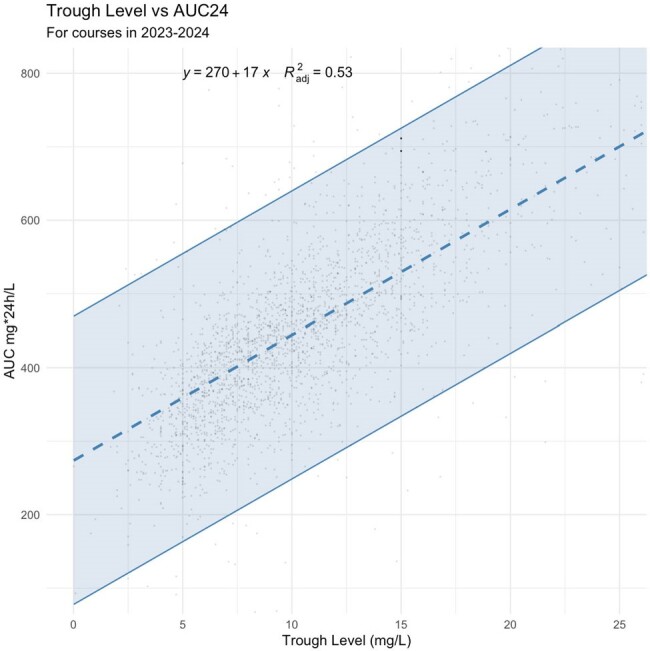

Prediction interval for AUC at any given trough

**Disclosures:**

**Marc H. Scheetz, PharmD, MSc**, Abbvie: Advisor/Consultant|Basilea: Advisor/Consultant|Cidara: Advisor/Consultant|DoseMe: Advisor/Consultant|Entasis: Advisor/Consultant|F2G: Advisor/Consultant|GSK: Advisor/Consultant|Lykos: Advisor/Consultant|Roche: Advisor/Consultant|Third Pole Therapeutics: Advisor/Consultant|Xelia: Advisor/Consultant **Paul Sabourenkov, MSc(Adv)Bioinf**, DoseMe Pty Ltd: Employee **Sharmeen Roy, PharmD, BCPS**, DoseMe: Employee

